# Incidence and Risk Factors of Intraventricular Hemorrhage in Premature Infants at King Faisal Military Hospital, Southern Region: A Retrospective Review

**DOI:** 10.7759/cureus.81214

**Published:** 2025-03-26

**Authors:** Khalid F Alghadam, Mohammed Alomari, Abdulrahman A Almehery, Badriah G Alasmari, Muhammad Saeed

**Affiliations:** 1 Neonatal Intensive Care Unit, Armed Forces Hospital Southern Region, Khamis Mushait, SAU; 2 Pediatrics, Armed Forces Hospital Southern Region, Khamis Mushait, SAU

**Keywords:** intraventricular hemorrhage (ivh), ivh prevention, kingdom of saudi arabia (ksa), preterm neonate, retrospective cohort study

## Abstract

Background

Intraventricular hemorrhage (IVH) is a serious complication in preterm neonates. IVH risk factors include neonatal anemia, use of inotrope and hydrocortisone, and pulmonary hemorrhage. This study was conducted in the Southern Region of Saudi Arabia to assess IVH epidemiology in small and premature infants.

Method

This study is a systematic two-year retrospective examination of a cohort of preterm infants born in King Faisal Military Hospital in the Southern Region of Saudi Arabia. A hierarchical multiple logistic regression modeling of data was adopted to evaluate the impact of potential risk factors.

Results

The study included 133 medical records, with an IVH incidence of 21.8% (n = 29). Most IVH cases were grade II (34.5%). The risk of IVH was higher with increasing birth weight (odds ratio (OR) = 63.2, p = 0.019), use of acidosis medications (OR = 15.8), and thrombocytopenia (OR = 24.2, p = 0.000024). In contrast, a decrease in gestational age (GA) was associated with a higher risk of IVH (OR = 0.85, p = 0.000085). A statistically significant association was found between birth weight and small for gestational age (SGA) status (p = 0.0374). Among non-SGA infants, higher birth weight increased IVH risk, whereas higher birth weight in SGA infants reduced IVH risk.

Conclusion

IVH incidence is still high in Saudi Arabia among preterm newborn infants despite advances in the healthcare system. This study identified birth weight, use of acidosis medications, and thrombocytopenia as significant risk factors.

## Introduction

Over 15 million neonates are born prematurely across the globe every year [1]. Intraventricular hemorrhage (IVH) is a serious complication of premature newborn infants (<32 weeks with birth weight less than 1,500 g). IVH occurs when bleeding ensues secondary to the rupture of blood vessels within the germinal matrix tissue of the developing brain into the ventricular system. The severity of IVH ranges from grade I to grade IV, described as most severe. Among neonates weighing under 1,500 g, the estimate for IVH is 27% [2]. About 90% of cases of IVH occur within the first three days of life, and in 20%-40% of affected infants, the hemorrhage progresses within the first week [3]. The exact pathogenesis of IVH remains unclear [4]. Therefore, preventative measures cannot be effectively devised. Prognosis, hence, for IVH is difficult to predict [5].

IVH is associated with substantial morbidity and mortality. One of the notable complications of IVH is the so-called parenchymal hemorrhagic infarction and post-hemorrhagic ventricular dilation that often lead to death or serious neurodevelopmental impairment in affected infants [6]. In Saudi Arabia, IVH in preterm neonates is associated with a 55% risk for neurodevelopmental dysfunction by the age of five years [7]. Moreover, IVH has been closely associated with an increased risk of renal impairment among Saudi preterm neonates [8]. Medical treatment for IVH ranges from addressing the coagulation profile to controlling respiratory distress, whereas surgical management targets the diversion of cerebrospinal fluid through ventriculoperitoneal shunting and ultimately the reduction of the burden created by intraventricular clots [9].

In Saudi Arabia, the incidence of IVH remains quite high despite advanced healthcare services provided at all levels of obstetric and pediatric neonatal settings. In a recent survey of over 100 premature infants from Riyadh, a range of risk factors were identified for severe IVH [10]. These included neonatal anemia, use of inotrope and hydrocortisone to neonates, pulmonary bleeding, non-use of antenatal steroids or dexamethasone, pulmonary hemorrhage, and presence of patent ductus arteriosus (PDA) deformity. Management of IVH in premature newborn infants in Saudi Arabia, as elsewhere, remains a considerable challenge with a range of supportive surgical measures failing to achieve desired outcomes [11]. Moreover, many medical interventions are associated with severe adverse effects [12]. The three-year mortality rate for IVH in Saudi Arabia is about 27.9% [13]. Despite studies reporting IVH incidence and risk factors in Saudi Arabia, limited research is available from the Southern Region of Saudi Arabia. Given the high mortality rate associated with IVH in Saudi Arabia, there is a need for research to assess IVH incidence and risk factors in the Southern Region of Saudi Arabia, which is economically disadvantaged compared to other parts of the country. Previously, most studies of IVH incidence have been conducted from the central region [7,10].

We conducted this study in the Southern Region of Saudi Arabia to assess the incidence and risk factors of IVH in premature infants equal to or less than 32 weeks with birth weight equal to or less than 1,500 grams.

## Materials and methods

Study design and setting

This was a retrospective cohort study based on a review of medical records. The study was conducted at King Faisal Military Hospital, Southern Region of Saudi Arabia, a tertiary care hospital with a specialized neonatal intensive care unit (NICU). The study included data from premature infants born between January 2020 and December 2021. The two-year study period was selected to capture a representative cohort of premature infants, ensuring sufficient data to assess the incidence and risk factors for IVH in this population.

Study population

All preterm infants born at or before 32 weeks of gestational age (GA) with a birth weight of 1,500 g or less were included. Infants were excluded if they had severe congenital anomalies or if they did not survive beyond 72 hours after birth. Small for gestational age (SGA) was defined as infants with a birth weight less than the 10th percentile for their gestational age, based on standard growth charts.

Sampling technique

A consecutive sampling method was used to include all eligible preterm infants born within the study period. Data was systematically extracted from electronic and paper-based medical records and entered into an anonymized Microsoft Excel spreadsheet (Microsoft Corp., Redmond, WA) for analysis. Missing data were handled by imputation based on available information where applicable, with records excluded from analysis if key variables were missing.

Data collection

The data collection process involved extracting demographic, clinical, and laboratory parameters relevant to the development of IVH. Information included gestational age, birth weight, sex, mode of delivery, use of antenatal steroids, presence of SGA status, perinatal asphyxia, inotropic support, acidosis management, ventilation support, high-frequency oscillatory ventilation (HFOV), coagulation status, thrombocytopenia, patent ductus arteriosus (PDA), surfactant administration, and pneumothorax. IVH diagnosis and grading were based on cranial ultrasound findings recorded in the medical records.

Ethical considerations

Approval for the study was obtained from the Institutional Review Board (IRB) and the Local Research and Ethics Committee. Patient confidentiality was strictly maintained, with all data anonymized before analysis.

Statistical analysis

Descriptive and inferential statistical analyses were conducted using SPSS software (IBM Corp., Armonk, NY). Continuous variables were summarized using means and standard deviations (SDs), while categorical variables were expressed as frequencies and percentages. Comparisons between infants with and without IVH were performed using the chi-square (χ2) test for categorical variables and independent t-tests for continuous variables. A penalized logistic regression model was used for multivariable analysis, identifying independent risk factors for IVH. Adjusted odds ratios (ORs) with 95% confidence intervals (CIs) were reported. Additionally, an interaction term was included between birth weight and SGA status to assess their combined effect on IVH risk. A significance threshold of p < 0.05 was used throughout the analysis.

## Results

**Figure 1:**
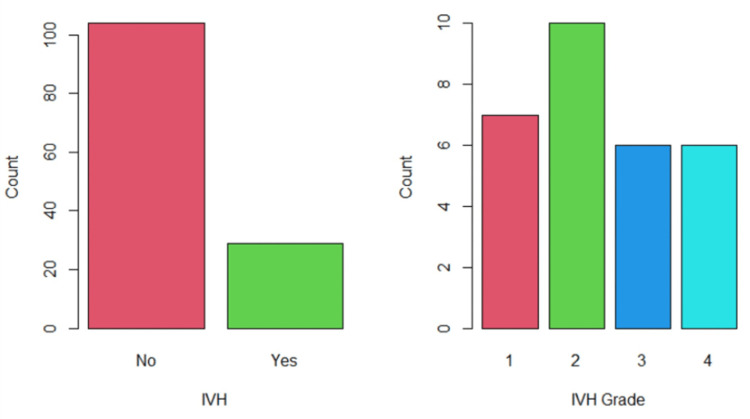
Estimated IVH incidence and its grades IVH: intraventricular hemorrhage

The study included 133 preterm infants. The incidence of IVH was 21.8% (n = 29) in premature infants (95% confidence interval (CI): 21.6%-22.1%). Grade I IVH incidence was 24.1% (n = 7), grade II was 34.5% (n = 10), and grade III and grade IV were both 20.7% (n = 6) infants each. A visual display of IVH incidence and IVH grades is provided in Figure 1.

**Figure 2:**
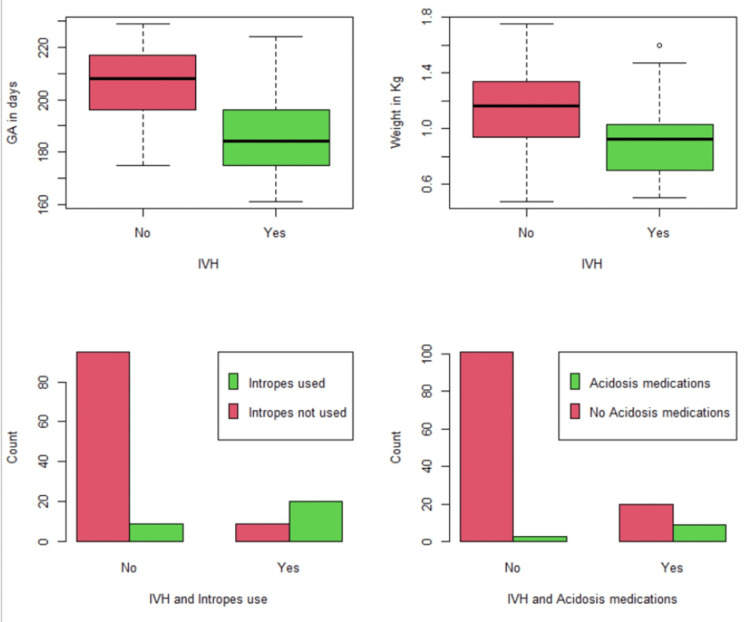
Significant effect for GA, birth weight, use of inotropes, and acidosis medications on IVH incidence Figure 2 shows, at the top, two box-and-whisker plots for the distribution of GA and birth weight broken down according to IVH cases. Mean GA and mean birth weights are clearly lower among IVH infants. The bottom figures show that the percentages of patients needing inotropes and acidosis medications are far higher in IVH than in non-IVH cases. IVH: intraventricular hemorrhage, GA: gestational age

**Figure 3:**
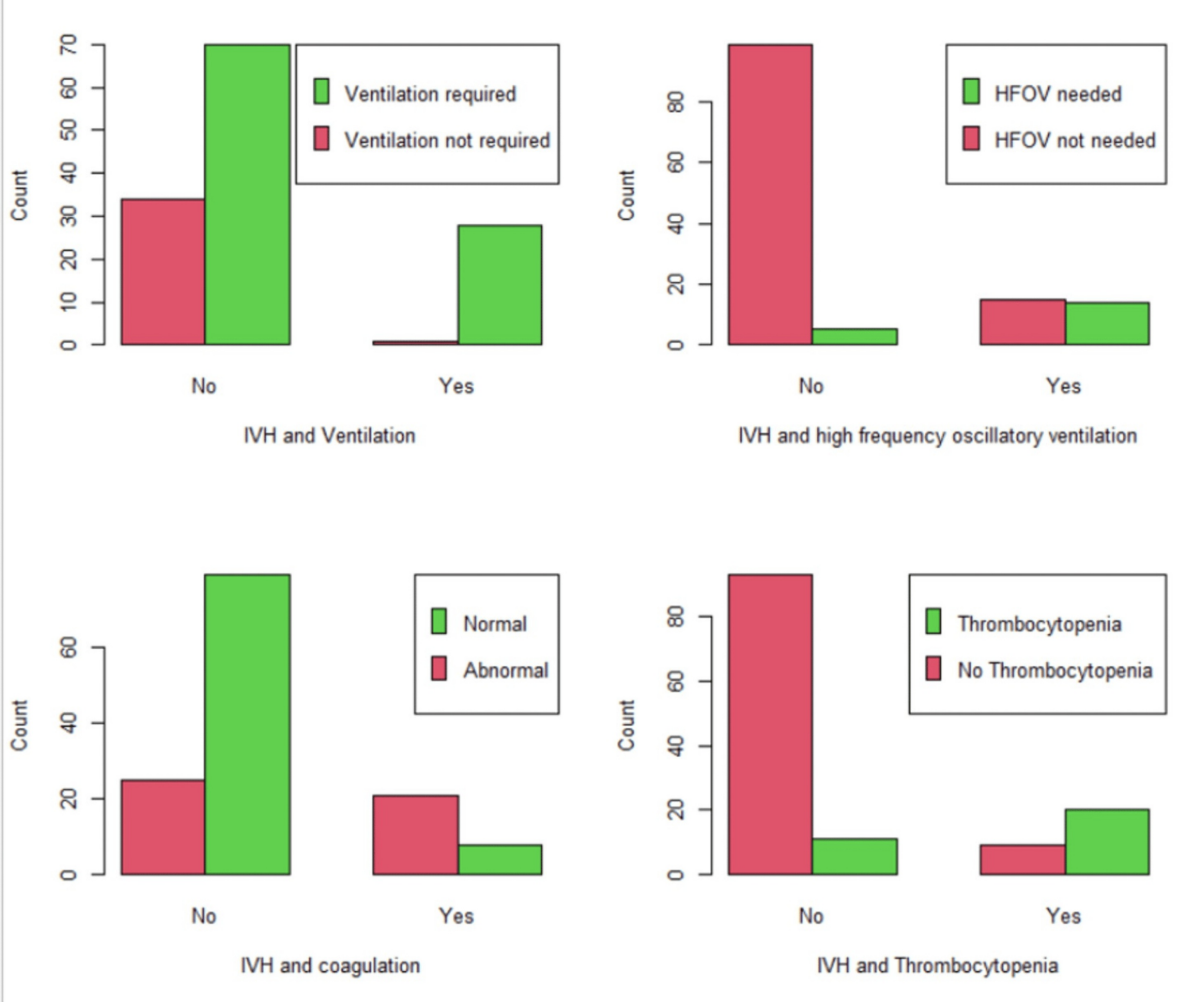
Significant effects of use of ventilation, HFOV, coagulation profile, and thrombocytopenia on IVH incidence Figure 3 shows that, at the top left, the ventilation percentage among IVH (96.6%) exceeds that among non-IVH cases (χ2(1) = 8.550, p = 0.0035). On the top right, HFOV was required (n = 14, 48.3%) of all IVH, compared to non-IVH cases (χ2(1) = 31.532, p < 0.0001). The bottom two figures show that abnormal coagulation and low platelets are far more prevalent among IVH cases (χ2(1) = 21.367, p < 0.0001 and χ2(1) = 40.044, p < 0.0001, respectively). IVH: intraventricular hemorrhage, HFOV: high-frequency oscillatory ventilation

Participants' demographic results are displayed in Table 1 alongside their unadjusted association with IVH. A visual display of the factors that significantly impacted IVH diagnosis is provided in Figure 2 and Figure 3.

**Table 1:** Demographic characteristics of the study participants and their unadjusted effect on IVH incidence χ2(1): chi-square (degree of freedom), BW: birth weight, SD (σ): standard deviation, (µ): mean, IVH: intraventricular hemorrhage, LBW: low birth weight, VLBW: very low birth weight, ELBW: extremely low birth weight, SGA: small for gestational age, IU: intrauterine, HFOV: high-frequency oscillatory ventilation, PDA: patent ductus arteriosus

Factor	Count (number)	Mean±SD/percentage	IVH prevalence	Test statistic	P-value
Gestational age (weeks)	Overall: 133	29±5 weeks 5 days			
	IVH: 29	27 weeks		t = 5.558	<0.0001
	Non-IVH: 104	29 weeks 4 days			
BW (kg)	Overall: 133	1.094±0.277 kg			
	IVH: 29	0.944 kg		t = 3.119	0.0034
	Non-IVH: 104	1.135 kg			
BW category					
LBW	9	6.8%	1 (3.4%)	χ²(2) = 4.169	0.1243
VLBW	72	54.1%	12 (41.4%)		
ELBW	52	39.1%	16 (55.2%)		
Mode of delivery					
Cesarean section	106	79.7%	21 (72.4%)	χ²(1) = 0.709	0.3998
Vaginal	27	20.3%	8 (27.6%)		
SGA					
Yes	37	27.8%	6 (20.7%)	χ²(1) = 0.540	0.4626
No	96	72.2%	23 (79.3%)		
Sex					
Female	67	50.4%	13 (44.8%)	χ²(1) = 0.217	0.6414
Male	66	49.6%	16 (55.2%)		
Steroids					
Yes	86	64.7%	17 (58.6%)	χ²(1) = 0.302	0.5824
No	47	35.3%	12 (41.4%)		
Asphyxia					
Yes	1	0.8%	0 (0%)	χ²(1) = 0	1
No	132	99.2%	29 (100%)		
IU infection					
Yes	7	5.3%	2 (6.9%)	χ²(1) = 0	1
No	126	94.7%	27 (93.1%)		
Inotropes					
Yes	29	21.8%	20 (69%)	χ²(1) = 44.906	<0.0001
No	104	78.2%	9 (31%)		
Acidosis medications					
Yes	12	9%	9 (31%)	χ²(1) = 18.596	<0.0001
No	121	91%	20 (69%)		
Ventilation					
Yes	89	73.7%	28 (96.6%)	χ²(1) = 8.550	0.0035
No	35	26.3%	1 (3.4%)		
HFOV					
Yes	19	14.3%	14 (48.3%)	χ²(1) = 31.532	<0.0001
No	114	85.7%	15 (51.7%)		
Coagulation					
Normal	87	65.4%	8 (27.6%)	χ²(1) = 21.367	<0.0001
Abnormal	46	34.6%	21 (72.4%)		
Thrombocytopenia					
Yes	31	23.3%	20 (69%)	χ²(1) = 40.044	<0.0001
No	102	76.7%	9 (31%)		
PDA					
Yes	100	75.2%	26 (89.7%)	χ²(1) = 3.228	0.0723
No	33	24.8%	3 (10.3%)		
Surfactant					
Yes	99	74.4%	27 (93.1%)	χ²(1) = 5.595	0.0180
No	34	25.6%	2 (6.9%)		
Pneumothorax					
Yes	13	9.8%	6 (20.7%)	χ²(1) = 3.5525	0.05946
No	120	90.2%	23 (79.3%)		

The mean gestational age was 29 weeks (±SD σ = 5 weeks 5 days), ranging between 23 weeks and 32 weeks. The median gestational age was 28 weeks 6 days. The mean gestational age in those infants who developed IVH was 27 weeks, significantly lower than the mean 29 weeks 4 days in those infants who did not develop IVH (t = 5.558, p < 0.0001).

The mean birth weight was 1.094 kg (±SD σ = 0.277 kg), ranging between 0.475 kg and 1.465 kg. The median birth weight was 1.050 kg. The mean birth weight in those infants who developed IVH was 0.944 kg, significantly lower than the mean weight of 1.135 kg in those infants who did not develop IVH (t = 3.119, p = 0.0034).

Inotropes were used in 21.8% (n = 29) of infants but were significantly associated with 69% (n = 20) of cases of all IVH (χ2(1) = 44.906, p < 0.0001). Moreover, acidosis medications were used in 9% (n = 12) of surveyed infants' records but were significantly associated with 31% (n = 9) of cases of all IVH (χ2(1) = 18.596, p < 0.0001). Furthermore, ventilation was needed in 73.7% (n = 89) of infants but was significantly associated with 96.6% (n = 28) of IVH cases (χ2(1) = 8.550, p = 0.0035). HFOV was required in 14.3% (n = 19) of premature infants and was associated significantly with 48.3% (n = 14) of all IVH (χ2(1) = 31.532, p < 0.0001). Abnormal coagulation profile defined as any deviation from the normal range of clotting parameters (such as prolonged prothrombin time or activated partial thromboplastin time, low fibrinogen levels, or abnormal platelet function) was noted among 34.6% (n = 46) of premature infants but was associated significantly with 72.4% (n = 21) of IVH incidents (χ2(1) = 21.367, p < 0.0001). Similarly, thrombocytopenia was observed in 23.3% (n = 31) of premature infants; however, it was associated with 69% (n = 20) of all IVH cases (χ2(1) = 40.044, p < 0.0001).

**Figure 4:**
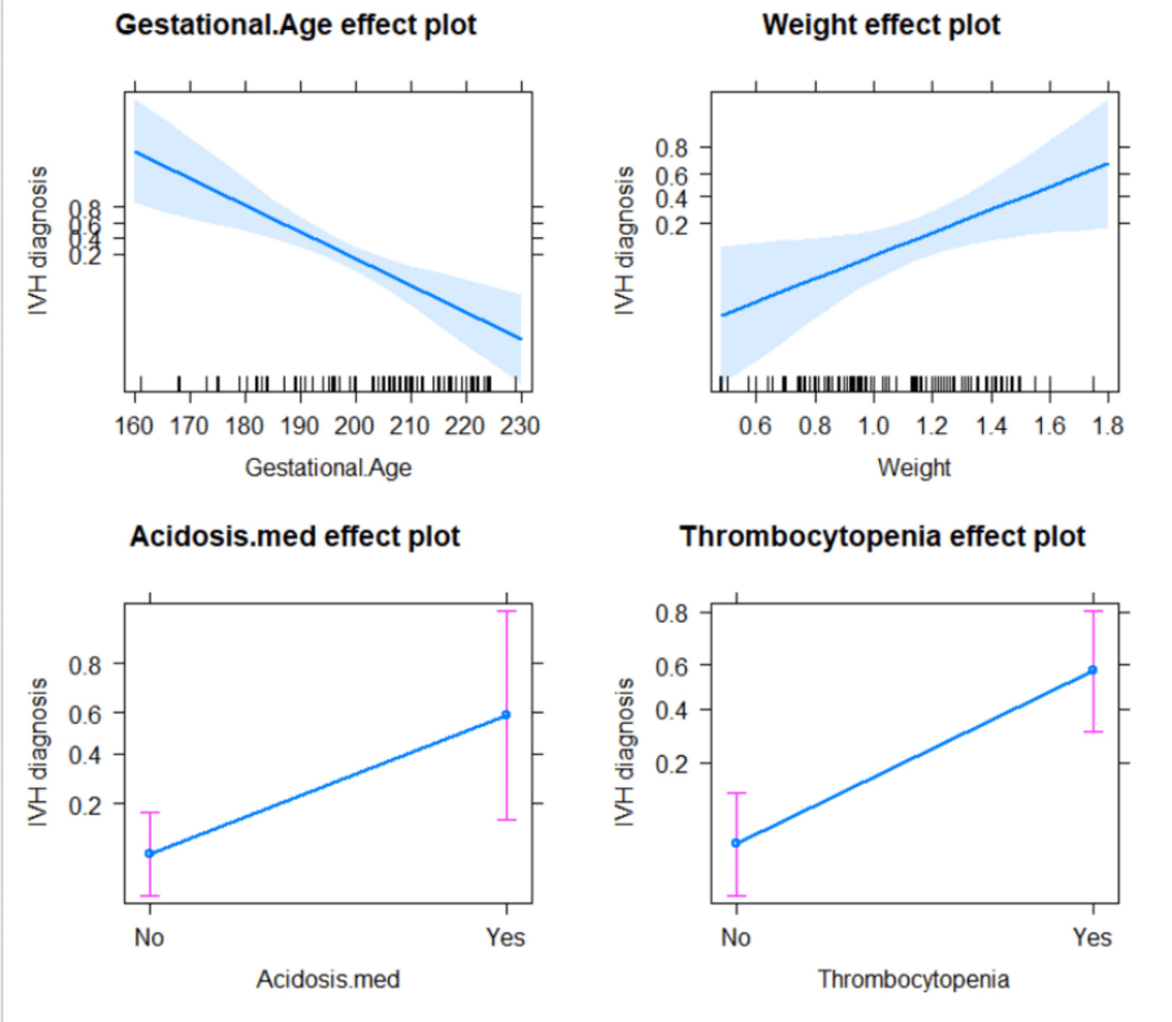
Adjusted effects of background factors on IVH among participating premature infants At the top left figure, as gestational age increases, the incidence of IVH drops, whereas, on the top right graph, as birth weight increases, the IVH incidence increases. The top graphs indicate clearly that patients needed acidosis medications and patients with low platelets are far more susceptible to IVH risk. IVH: intraventricular hemorrhage

Table 2 gives a detailed account of the adjusted effects for the hierarchical logistic regression model for factors that were significantly associated simultaneously with IVH incidence. It was notable that IVH incidence was increased, at the adjusted level, as birth weight increased (OR = 63.1978, p = 0.019134), as we prescribed acidosis medication (OR = 15.7916), and with thrombocytopenia (OR = 24.1859, p = 0.000024). However, as gestational age decreases, i.e., the more premature an infant is, the more likely they will develop IVH (OR = 0.8521, p = 0.000085) (Figure 4).

**Table 2:** Impact of background factors on IVH incidence among the medical records examined as selected in the final hierarchical model * p < 0.05 (statistically significant), *** p < 0.001 (very highly significant) IVH: intraventricular hemorrhage, OR: odds ratio, CI: confidence interval

Factor	OR	95% CI of OR	P-value
Gestational age	0.8521	0.7867-0.9229	0.000085***
Birth weight	63.1978	1.9692-2028.1952	0.019134*
Acidosis medications	15.7916	1.6705-149.2818	0.016053*
Thrombocytopenia	24.1859	5.5146-106.0742	0.000024***

**Figure 5:**
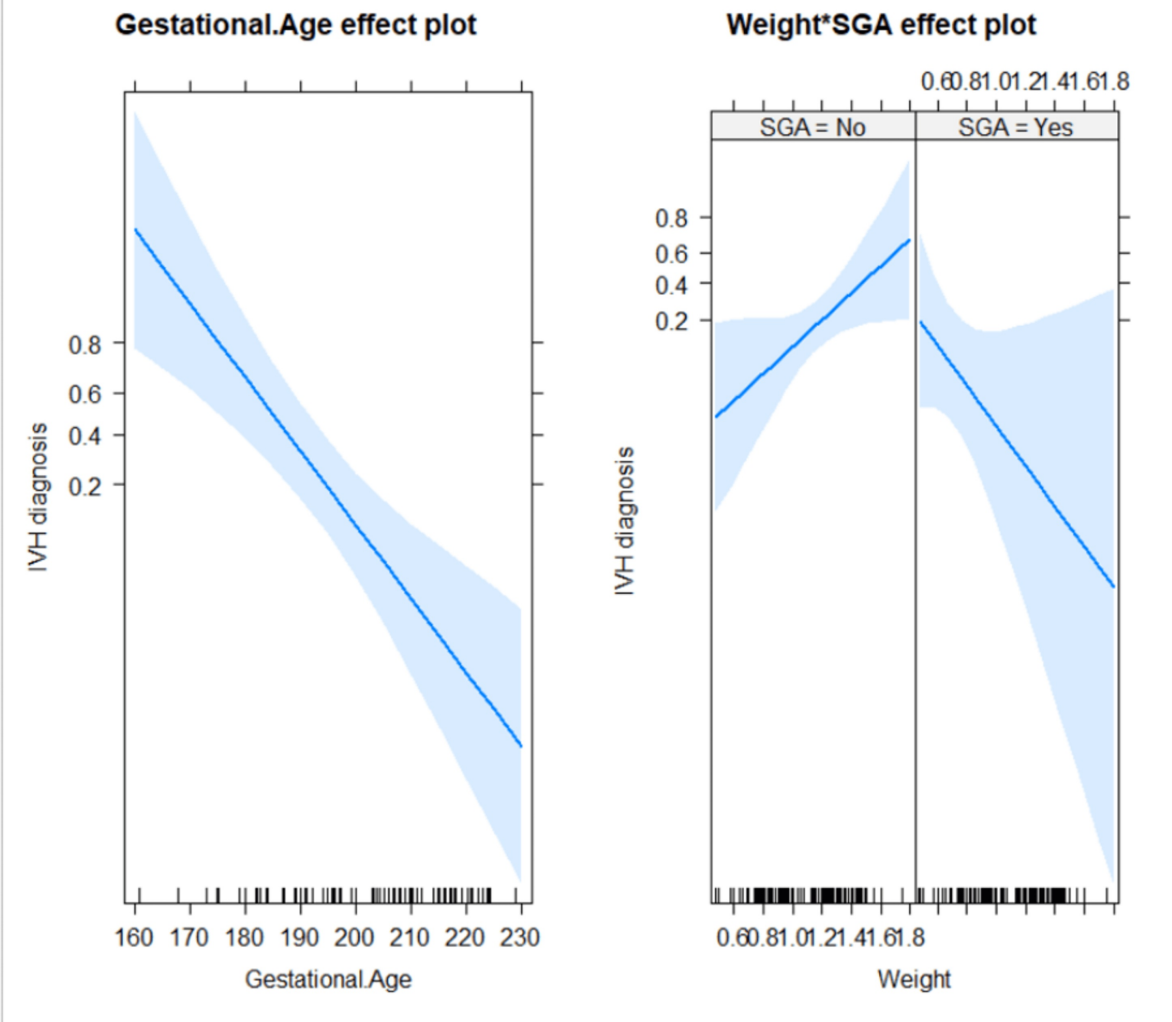
Interaction between birth weight and SGA adjusted for gestational age on IVH among participating premature infants The right graph shows clearly that significant interaction existed between birth weight and SGA status (p = 0.0374), i.e., in patients with no SGA status, birth weight increased IVH risk; however, among SGA infants, higher birth weight was associated with reduced risk for IVH. IVH: intraventricular hemorrhage, SGA: small for gestational age

To further understand the effect of birth weight, we included an interaction term between birth weight and SGA in the model adjusted for gestational age (Table 3 and Figure 5).

**Table 3:** Interaction between birth weight and SGA adjusted for gestational age on IVH among participating premature infants * p < 0.05 (statistically significant), *** p < 0.001 (very highly significant) IVH: intraventricular hemorrhage, SGA: small for gestational age, OR: odds ratio, CI: confidence interval

Factor	OR	95% CI of OR	P-value
Gestational age	0.8649	0.8089-0.9249	0.00002***
Birth weight	37.5660	1.1944-1181.4810	0.0393*
SGA	1024.8484	1.0383-1011547.6068	0.0488*
Birth weight × SGA interaction	0.0001	0-0.5897	0.0374*

There was a statistically significant interaction between birth weight and SGA status (p = 0.0374), i.e., in patients with no SGA status, birth weight increased the IVH risk; however, among SGA infants, higher birth weight was associated with reduced risk for IVH.

## Discussion

The current study systematically investigated a large sample of medical case notes related to a two-year cohort of premature infants born in King Faisal Military Hospital in the Southern Region of Saudi Arabia.

Our results showed that the incidence of IVH is 21.8%, mostly grade II IVH (24.1%). This is slightly lower than the overall IVH incidence of 27.1% reported by Allen [2] but within earlier estimates of 20%-25% [14]. In a very recent Saudi survey for severe IVH, the incidence was estimated to be 10.8% [15], which is comparable to the 12.9% incidence we found for IVH grades III and IV combined in our cohort. However, we are confident that our overall incidence estimate lies within the global incidence estimates that range between 5% and 52% [16]. Notably, our study spans a two-year duration; however, we may not be able to capture trends in IVH incidence that could be operating over the last 20-50 years. Recent investigations indicated a rising trend in IVH [17]. This increasing trend in IVH among preterm infants could also be the case in Saudi Arabia [4]. Research centers in Saudi Arabia would benefit greatly from setting up data observatories that focus on identifying trends in IVH over prolonged periods of time.

We found that at the adjusted level, risk factors for IVH among newborn preterm infants include birth weight, use of medications to treat acidosis, and thrombocytopenia. Expectedly, we found that gestational age reduces the risk for IVH among our cohort. This remains a consistent finding across studies on risk factors for IVH [4]. IVH is almost linearly far more prevalent in extremely premature infants with younger gestational age [6,14]. Gestational age has a significant impact on angiogenesis and brain organization. The connection between impaired angiogenesis and altered neurogenesis is well-established [18]. A low gestational age is associated with impaired expression of astrocyte structure, altered tight junction development, and defective neurological basal membrane development [19]. Furthermore, IVH-related bleeding halts cell proliferation and results in long-term adverse effects on both astrocytogenesis and oligogenesis [20]. Our results show clearly that in Saudi Arabia, infants with smaller gestational ages at birth were at exceedingly higher risk of developing IVH.

An unexpected finding in our study was the positive association between higher birth weight and an increased likelihood of IVH risk when adjusted for other factors. At the unadjusted level, birth weight was intuitively associated with a reduction in IVH risk, but this pattern reversed after adjustment for confounding variables. This contradicts the established literature that low birth weight is a recognized predictor for IVH [21]. One potential explanation for this finding could be statistical confounding or the interaction between birth weight and other variables, such as SGA status. At the adjusted level, the effect of birth weight might be influenced by other underlying factors not fully accounted for in the unadjusted analysis. However, as we focused the analysis on SGA interaction with birth weight, it emerged clear that a more intuitive connection occurred at the SGA level. Among our cohort, infants with SGA status were more likely to develop IVH when they had smaller birth weights. That was a unique finding from our investigation and merits further research for a more focused evaluation.

Our findings indicate that patients who developed IVH were in greater need of acidosis medications. This particular finding is in agreement with mainstream literature. Acidosis was one of the consistent risk factors reported to increase the risk for IVH among premature newborn infants [22]. Furthermore, acidosis is associated with mortality in this age group of patients as well as increased risk for various neurodevelopmental disorders. It is postulated that acidosis directly affects hemostatics by affecting how platelets function and preventing their effective aggregation [23].

We noted a significant association between low platelet count and risk of IVH. However, such an association was not a consistent finding in recent studies. In one survey, although preterm infants with IVH were found to need more units of blood transfusion, platelet count was not different from infants without IVH [24]. On the other hand, as per our results, some surveys indicated an increased IVH hazard (almost double) with thrombocytopenia, with a higher need for platelet transfusion [25,26]. The debate of whether thrombocytopenia is a recognized risk factor for the development of IVH in preterm newborn babies is likely to remain unsolved for some time. Our current study results, nonetheless, support the connection between increased IVH risk and low platelet count.

Delivery mode, through cesarean section (CS) or vaginal route, did not significantly impact IVH risk among our cohort. We, thus, confirm an established theory that delivery mode does not influence IVH development risk [27]. However, some recent investigations indicated a protective effect for CS against IVH [28]. In Saudi Arabia, a single study indicated reduced IVH risk among infants born through the CS route [29]. Clearly, in light of such conflicting results, further robust research is required to help reconcile these findings.

We could not identify intrauterine infection as a significant risk factor for the development of IVH. Recent investigations noted that chorioamnionitis may be a greater risk factor for prematurity rather than directly for IVH [30]. This could explain our findings. However, future studies should follow patients with intrauterine infection longitudinally and adopt robust statistical modeling methods that take account of the interaction effect between prematurity, IVH, and infection.

Strengths and limitations of the study

A range of strengths can be identified in the current study. The longitudinal systematic cohort design and robust statistical analysis are two main strengths of our investigation. However, we relied primarily on medical records; therefore, first-hand diagnostic input is missing from our data set. Any source-related transcription clinical errors or missing data will inevitably affect our results. Another limitation of the study was the small sample size. Although our study found a significant association between birth weight and IVH, the retrospective design and modest sample size limit our ability to draw firm causal inferences. The observational nature of the study means that, despite statistical adjustments, there may be residual confounding from variables we were unable to measure or fully control. Additionally, the small sample size reduces the statistical power to detect subtle effects and increases the likelihood that chance findings or measurement errors could influence our results. Future prospective studies with larger cohorts are necessary to validate these associations and more definitively explore causality.

Clinical implications and future directions

The findings of this study have important clinical implications for the care of premature infants. Understanding the key risk factors, such as gestational age, birth weight, acidosis, and thrombocytopenia, can help clinicians better identify infants at higher risk for IVH. Future research should focus on prospective studies that examine the causal mechanisms behind the observed associations, particularly the relationship between birth weight, SGA status, and IVH risk. Longitudinal studies are needed to track the long-term effects of IVH on neurodevelopmental outcomes in preterm infants. Further exploration of additional risk factors, such as intrauterine infections and genetic predispositions, could provide a more comprehensive understanding of IVH development.

## Conclusions

IVH incidence is high in Saudi Arabia among preterm newborn infants despite advances in the healthcare system. In the present study, approximately one in five children had IVH. In the majority of cases, the severity of IVH was moderate. Increased risk for IVH is associated with birth weight, use of medications to treat acidosis, and thrombocytopenia. Gestational age reduces IVH risk. There is substantial interaction between SGA and birth weight in terms of effect on IVH risk. Early identification of high-risk infants through routine ultrasound examination can reduce IVH incidence. Optimizing perinatal management, including judicious use of inotropes and minimizing exposure to HFOV, could also play a role in preventing IVH. Future research should focus on prospective studies that examine the causal mechanisms behind the observed associations, particularly the relationship between birth weight, SGA status, and IVH risk.
